# An Unusual Case of Fulminant Type 1 Diabetes during the Second Trimester of Pregnancy

**DOI:** 10.1155/2014/968547

**Published:** 2014-08-11

**Authors:** Tomohiro Okuda, Sadao Yamashita, Yoshio Ogino, Hisashi Kataoka, Jo Kitawaki

**Affiliations:** ^1^Department of Obstetrics and Gynecology, Fukuchiyama City Hospital, 231 Atsunaka-cho, Fukuchiyama, Kyoto 620-8505, Japan; ^2^Department of Obstetrics and Gynecology, Graduate School of Medical Science, Kyoto Prefectural University of Medicine, Kyoto 602-8566, Japan

## Abstract

Fulminant type 1 diabetes is a new subtype of rapid-onset type 1 diabetes, with pancreatic exocrine dysfunction, that usually develops during the third trimester of pregnancy. We describe a patient with fulminant type 1 diabetes onset during her second trimester, resulting in premature delivery. The 34-year-old woman, without any known risk factors for diabetes mellitus, experienced a sudden stillbirth at 24-weeks gestation. Her blood glucose level was 950 mg/dL and she was positive for urine ketone bodies. The condition met all the diagnostic criteria for fulminant type 1 diabetes, and was diagnosed as such. Although this disease is rare, its progression is rapid, and its clinical course is severe and occasionally leads to death; therefore, a full knowledge of the disease is important to facilitate an accurate diagnosis.

## 1. Introduction

Fulminant type 1 diabetes is a new subtype of type 1 diabetes in which the pancreatic islet cells fail rapidly, leading to hyperglycemia and ketoacidosis; the incidence of this disease clearly increases during the third trimester of pregnancy [1, 2]. Here, we describe a case of fulminant type 1 diabetes mellitus during the second trimester of pregnancy, which resulted in ketoacidosis and a premature birth.

## 2. Case Presentation

The patient was a 34-year-old Japanese woman (gravida 1, para 1) whose previous personal and family medical histories were unremarkable. During this pregnancy, the patient's physical findings, cervical length, laboratory data, urinalyses, and fetal growth determinations were all within normal ranges; glucosuria or ketonuria was not detected.

At 23 weeks and 6 days of gestation, physical findings, fetal growth, cervical length, laboratory data, and urinalysis were all within normal range. Two days later, the patient thought she had caught a cold because of the onset of malaise and thirst. At 24 weeks and 3 days of gestation, she presented with complaints of nausea and lumbago. Upon examination, the fetal heartbeat and movement were positive. The physician on duty at the time of admission initially diagnosed the condition as poor physical condition due to the common cold. However, 5 h later, the patient went into labor, and the gestational sac and fetal head appeared, resulting in emergent hospitalization and a subsequent premature delivery.

Upon admission, the patient was 166 cm tall, weighing 72.7 kg (before pregnancy, 63 kg), and had a body temperature of 36.9°C and a blood pressure of 156/74 mmHg. A physical examination revealed labored breathing and cold extremities. A stillborn infant (592 g, female) was soon delivered. The associated blood loss was minimal, and uterine contraction was good; the placenta and umbilical cord (130 g) appeared normal. Soon after delivery, the patient's vitals were normal, apart from a blood pressure of 129/69 mmHg and a slight tachycardia, with a pulse of 113 beats/min. The patient had no lower abdominal pain but complained of epigastric pain.

The results of laboratory tests on blood collected during labor were examined (Table 1) and confirmed an extreme hyperglycemia, with a blood glucose level of 950 mg/dL. Her urinary sugar and ketones were markedly elevated. The labored breathing observed during delivery became more severe, and the patient developed clouded consciousness, leading to management in the intensive care unit (ICU).

**Figure 1:**
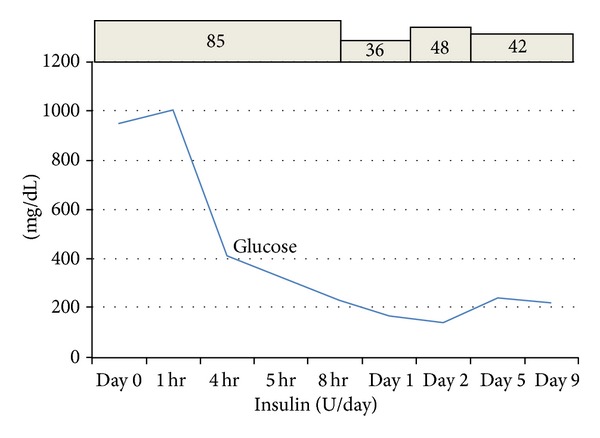
The variations in the blood glucose levels after admission. The patient's laboratory blood glucose levels improved with this treatment.

**Figure 2:**
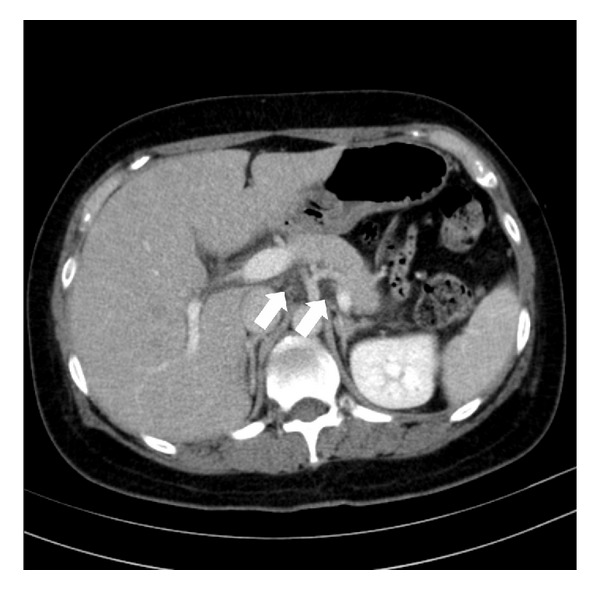
Abdominal computed tomography (CT) scan taken 24 h after admission. The image shows a mild enlargement of the pancreas and a diffuse inflammation of the peripancreatic tissues (⇒); however, there was no exudate. There was no pancreatic necrosis. The CT severity index is 2.

After admission, the patient's white blood cell count, serum amylase levels, blood urea nitrogen levels (BUN), and blood creatinine levels were elevated, leading to a diagnosis of sepsis, acute renal failure, and acute pancreatitis associated with diabetic acidosis. Following a diagnosis of coma due to diabetic ketoacidosis (DKA), acute renal failure, acute pancreatitis, and sepsis, insulin therapy (regular insulin, 7 U/h) and antimicrobial therapy (meropenem trihydrate) were initiated in conjunction with adequate hydration. The patient's laboratory findings and blood gas data improved with this treatment (Figure 1), and she regained consciousness 24 h after admission to the ICU. Abdominal computed tomography (CT) was performed, revealing a CT severity index of 2 (Figure 2).

Despite elevated postadmission blood glucose levels, the patient's glycated hemoglobin (HbA1c) levels remained normal. However, C-peptide blood levels of 0.1 ng/mL indicated depleted insulin levels; antibodies against insulin, glutamic acid decarboxylase (GAD), and islet antigen-2 were not detected. Despite marked elevations of C-reactive protein and white blood cell levels, the patient's blood cultures were negative and she was afebrile. A search for factors contributing to DKA involved two investigations of possible viral infection (Table 2). The findings revealed elevated Coxsackie A9, B1, and B3 antibody titers. With the patient's consent, human leukocyte antigen (HLA) typing was performed; the findings showed HLA-DRB1∗0405-DQB1∗0401. The condition met all the diagnostic criteria of fulminant type 1 diabetes [2] and was diagnosed as such.

The patient was discharged 13 days postpartum and her insulin levels remain depleted, 1 year later. The patient has required regular insulin (42 U/day) administration, with her blood glucose levels varying within the range of 150–250 mg/dL; occasional hypoglycemic episodes preclude higher insulin doses. The patient's HbA1c levels have recently been maintained at approximately 8%.

## 3. Discussion

Buschard et al. previously reported patients with peripheral blood lymphocytes ratio changes, resembling active autoimmunity, 2 months before childbirth. The risk of developing type 1 diabetes during late pregnancy, therefore, increased 3.8-fold due to the involvement of autoimmune mechanisms [3]. Later, in 2000, Imagawa et al. reported that acute-onset type 1 diabetes mellitus could be classified into three subtypes, namely, the autoimmune type, nonautoimmune chronic type, and nonautoimmune fulminant type [1] and that the nonautoimmune fulminant type 1 diabetes develops in late pregnancy or during the puerperal period [4]. Initially, Imagawa et al. used the term “nonautoimmune fulminant type 1 diabetes” because of the inability to detect autoantibodies; however, since the cause of the subtype was unknown, the Japan Diabetes Society proposed the name “fulminant type 1 diabetes” [2]. In 2004, two sets of criteria were established for the diagnosis of the condition, namely, the “screening criteria" and the “diagnostic criteria.”

Fulminant type 1 diabetes is believed to develop due to an interaction between genetic and environmental factors [5]. Most cases are accompanied by symptoms of upper respiratory tract infections, suggesting that viral infections might be involved [5]. In 2005, Imagawa et al. reported that enterovirus infections could be a trigger for fulminant type 1 diabetes [6], and, in 2009, Tanaka et al. reported that enterovirus infections caused the devastating destruction of pancreatic beta cells, mediated by the chemokine circuit [7]. Genetically, HLA, a human histocompatibility complex molecule, is strongly involved [5]. Shimizu et al. reported that HLA-DRB1∗0405-DQB1∗0401 was involved as a trigger for fulminant type 1 diabetes not associated with pregnancy and that HLA-DQA1∗0302, 0501 and HLA-DRB1∗0301 (DR3), 0901 were involved in fulminant, pregnancy-associated type 1 diabetes [8]. However, in 2012, the Japan Diabetes Society revised the diagnostic criteria for fulminant type 1 diabetes and added HLA DRB1∗04:05-DQB1∗04:01 as a reference finding in the diagnostic criteria [2]. The present case met all of the diagnostic criteria for this disease. Specifically, the patient had a genetic predisposition for fulminant type 1 diabetes and demonstrated an antiviral immune response that might have caused destruction of the patient's pancreatic beta cells.

In recent years, fulminant type 1 diabetes has been reported among Asians [9, 10], South Americans [11], and Caucasians [12, 13]; however, the reported HLA prototypes are not always HLA-DRB1∗0405-DQB1∗0401. The incidence of fulminant type 1 diabetes among Japanese women is less than 10% of that found in Europeans and Americans; however, several reports on fulminant type 1 diabetes are from Japan [14, 15].

Treatment of DKA includes aggressive volume replacement, insulin infusion, and careful electrolyte monitoring. However, the most common complications of DKA include hypoglycemia due to overzealous insulin treatment, hypokalemia due to insulin administration, and bicarbonate treatment of acidosis [16]. Insulin lowers serum potassium; therefore, potassium supplementation should be maintained in intravenous fluids, as described above, and carefully monitored. If the serum potassium level is <3.3 mmol/L, potassium replacement therapy should be started immediately with fluid therapy, and the initiation of insulin therapy should be delayed until the potassium concentration is restored to >3.3 nmol/L to avoid arrhythmia, cardiac arrest, and respiratory muscle weakness. Bicarbonate therapy is the most controversial area in the treatment of DKA. At a pH >7.0, reestablishing insulin activity blocks lipolysis and resolves ketoacidosis without added bicarbonate. In addition, the use of insulin causes ketone body metabolism, leading to bicarbonate recovery; therefore, bicarbonate must not be used unless the pH is ≤ 7. The intravenous infusion of large amounts of bicarbonate is likely to cause a metabolic alkalosis, which is difficult to treat and is likely to cause respiratory depression that may lead to CO_2_ narcosis.

In addition, fulminant type I diabetes aggravates both the maternal and fetal prognoses [17]. When gestational diabetes is left untreated, there is a risk of developing obstetrical complications; therefore, glycemic control before and during pregnancy is important [18]. However, predicting and preventing the onset of fulminant type 1 diabetes is not possible with conventional glycemic control methods. The patient should be interviewed and asked about experiencing marked xerostomia, polydipsia, or polyuria. If acknowledged, urinalyses should be conducted to detect urinary glucose and urinary ketones. If present, verification of whether or not the condition is DKA is required. If DKA develops during pregnancy, the maternal mortality is 4–15% [19]. Additionally, respiratory compensation aimed at correcting metabolic acidosis should not be mistaken for a hyperventilation syndrome.

Although there have been numerous findings on type 1 diabetes and its subtype, fulminant type 1 diabetes, the pathogenesis of the latter remains unknown. However, gynecologists and obstetricians need to be aware that fulminant type 1 diabetes may develop during pregnancy. Healthy, pregnant women develop the condition with a sudden onset, accompanied by prodromes consisting of flu-like symptoms or acute gastroenteritis-like symptoms. If an accurate diagnosis and treatment are not promptly achieved, the condition may lead to death within a few days.

## Figures and Tables

**Table 1:** Laboratory findings upon admission.

Urinalysis	Ketone body 3+, glucose 4+

Arterial blood gas analysis	pH 6.98, PaO_2_: 129.0 mmHg, and PaCO_2_: 9.3 mmHg

HCO_3_^2^	1 mmol/L, base excess −27.6 mmol/L

Complete blood count	WBC: 32,050/*μ*L; hemoglobin: 13.6 g/dL; hematocrit: 41.5%; platelets: 37.9 × 10^4^/*μ*L; glucose: 950 mg/dL; HbA1c: 5.7%

Biochemistry	TP: 8.4 g/dL; albumin: 4.5 g/dL; GOT: 32 IU/L; GP: 23 IU/dL; LDH: 478 IU/L; gamma-GTP: 13 IU/L; amylase: 709 IU/L; CRP: 5.03 mg/dL; BUN: 37 mg/dL; creatinine: 1.58 mg/dL; Na: 128 mEq/L; K: 5.5 mEq/L; and Cl: 88 mEq/L

Coagulation	PT: 22.8 seconds; APTT: 42.4 seconds; INR: 1.20 seconds; Fibrinogen: 457 mg/dL; D-dimers: 106.3 *μ*g/mL

Antibodies	Insulin antibodies <125.0 nU/mL; GAD antibodies <1.3 U/mL; IA-2 antibodies <0.4 U/mL; C-peptide 0.1 ng/mL (2 h after meal)

Note: WBC: white blood cells; CRP: C-reactive protein; TP: total protein; GOT: glutamate-oxaloacetate transaminase; GPT: glutamate pyruvate transaminase; LDH: lactic dehydrogenase; gamma-GTP: gamma-glutamyl transpeptidase; BUN: blood urea nitrogen; PT: prothrombin time; APTT: activated partial thromboplastin time; INR: international normalized ratio; PaO_2_: partial oxygen pressure; PaCO_2_: partial carbon dioxide pressure; GAD: glutamic acid decarboxylase; IA-2: islet antigen-2.

**Table 2:** Variations in the levels of antibodies against Coxsackie viruses A9, B1, B2, B3, B4, B5, and B6 on the day of admission and at discharge (day 13).

Test	Day 0 (admission)	Day 13 (discharge)
Coxsackie A9 (NT)	8 Units	16 Units
Coxsackie B1 (NT)	16 Units	32 Units
Coxsackie B2 (NT)	<4 Units	<4 Units
Coxsackie B3 (NT)	32 Units	64 Units
Coxsackie B4 (NT)	<4 Units	<4 Units
Coxsackie B5 (NT)	<4 Units	<4 Units
Coxsackie B6 (NT)	<4 Units	<4 Units

NT: neutralization test.
